# Retraction: Perinatal Exposure to Bisphenol-A Impairs Spatial Memory through Upregulation of Neurexin1 and Neuroligin3 Expression in Male Mouse Brain

**DOI:** 10.1371/journal.pone.0220212

**Published:** 2019-07-18

**Authors:** The PLOS ONE Editors

Following publication of [1], the following concerns were raised:

- Figure 2A Nrxn1 panel (3 weeks) appears similar to Figure 2A Nrxn1 panel (8 weeks);
- Figure 2B GAPDH panel (3 weeks) appears similar to Figure 2A GAPDH panel (3 weeks);
- Figure 2B GAPDH panel (8 weeks) appears similar to Figure 2A GAPDH panel (8 weeks);
- Areas of similarity between Figure 3A BPA (3 weeks) and Figure 3B BPA (8 weeks);
- Similarity between Figure 3A Negative panels CC+DG at the 3 week and 8 week time points;
- Areas of similarity between Figure 5A DG+BPA (3 weeks) and Figure 5A DG+Sesame (8 weeks);
- Areas of similarity between Figure 5B DG+BPA (3 weeks) and Figure 5B DG+ Sesame (8 weeks);
- Areas of similarity between Figure 5B DG+Sesame (3 weeks) and Figure 5B DG+BPA (3 weeks);
- Areas of similarity between Figure 5B DG+Sesame (3 weeks) and Figure 5B DG+BPA (8 weeks);
- Areas of similarity between Figure 5B DG+BPA (3 weeks) and Figure 5B DG+Sesame (8 weeks) with Figure 4B DG+BPA (3 weeks) from a paper published in *Toxicology* by the authors [2].

For the RT-PCR experiment, authors noted that a representative image was chosen from three replicative experiments and provided all data underlying Figure 2A. The authors have explained that RT-PCR analysis of Nrxn1, Nlgn3 and GAPDH expression was carried out using the same cDNA preparation for both time points, therefore the GAPDH panels in Figure 2A and 2B are the same for both targets, resolving the concern raised.

For the experiments reported in Figure 3 and 5, authors provided some images and explained that they used the same image to illustrate negative controls in these experiments for both treatments at the 3 and 8 week timepoints. A member of our Editorial Board advised that the explanation and images provided do not resolve the concerns raised.

In light of the unresolved concerns that question the integrity of the findings, the *PLOS ONE* editors retract this article.

DK and MKT did not agree with retraction.
